# *Aniba rosaeodora* (Var. amazonica Ducke) Essential Oil: Chemical Composition, Antibacterial, Antioxidant and Antitrypanosomal Activity

**DOI:** 10.3390/antibiotics10010024

**Published:** 2020-12-30

**Authors:** Amanda Mara Teles, João Victor Silva-Silva, Juan Matheus Pereira Fernandes, Kátia da Silva Calabrese, Ana Lucia Abreu-Silva, Silvio Carvalho Marinho, Adenilde Nascimento Mouchrek, Victor Elias Mouchrek Filho, Fernando Almeida-Souza

**Affiliations:** 1 Pós-Graduação em Saúde do Adulto, Universidade Federal do Maranhão, 65080-805 São Luís, Brazil; damarateles@hotmail.com; 2 Laboratório de Imunomodulação e Protozoologia, Instituto Oswaldo Cruz, Fiocruz, 21040-900 Rio de Janeiro, Brazil; jvss2906@gmail.com (J.V.S.-S.); juan.fernandes222@gmail.com (J.M.P.F.); 3 Pós-graduação em Ciência Animal, Universidade Estadual do Maranhão, 65055-310 São Luís, Brazil; abreusilva.ana@gmail.com; 4 Laboratório de Óleos Essenciais, Universidade Federal do Maranhão, 65065-545 São Luís, Brazil; silviomarinho@yahoo.com.br; 5 Laboratório de Controle de Qualidade de Alimentos e Água, Universidade Federal do Maranhão, 65065-545 São Luís, Brazil; adenild@bol.com.br (A.N.M.); victor.mouchrek@ufma.br (V.E.M.F.)

**Keywords:** rosewood, linalool, marine bacteria, ABTS, *Trypanosoma cruzi*, cytotoxicity, nitrite, nitric oxide

## Abstract

*Aniba rosaeodora* is one of the most widely used plants in the perfumery industry, being used as medicinal plant in the Brazilian Amazon. This work aimed to evaluate the chemical composition of *A. rosaeodora* essential oil and its biological activities. *A. rosaeodora* essential oil presented linalool (93.60%) as its major compound. The *A. rosaeodora* essential oil and linalool showed activity against all the bacteria strains tested, standard strains and marine environment bacteria, with the lower minimum inhibitory concentration being observed for *S. aureus*. An efficient antioxidant activity of *A. rosaeodora* essential oil and linalool (EC_50_: 15.46 and 6.78 µg/mL, respectively) was evidenced by the inhibition of the 2,2-azinobis- (3-ethylbenzothiazoline-6-sulfonic acid) (ABTS) radical. The antitrypanosomal activity of *A. rosaeodora* essential oil and linalool was observed at high concentrations against epimatigote forms (inhibitory concentration for 50% of parasites (IC_50_): 150.5 ± 1.08 and 198.6 ± 1.12 µg/mL, respectively), and even higher against intracellular amastigotes of *T. cruzi* (IC_50_: 911.6 ± 1.15 and 249.6 ± 1.18 µg/mL, respectively). Both *A. rosaeodora* essential oil and linalool did not exhibit a cytotoxic effect in BALB/c peritoneal macrophages, and both reduced nitrite levels in unstimulated cells revealing a potential effect in NO production. These data revealed the pharmacological potential of *A. rosaeodora* essential oil and linalool, encouraging further studies.

## 1. Introduction

In recent decades, many studies have concentrated on the search for potential antimicrobials, with an increase in the worldwide spending on finding new antimicrobial agents. Faced with bacterial resistance to antibiotic treatment as well as the discovery of new pathogens, the need for new antimicrobials arises [1].

There are promising reports of different plant-derived natural phytochemicals, and a growing interest in exploring their potential [2]. Medicinal plants have been used around the world for various purposes, just as their active chemical compounds have been used to combat various diseases. Essential oils are well known for their pharmacological activities, including antibacterial [3] and trypanocidal activity [4], and may represent a promising source of new natural drugs.

**Figure 1:**
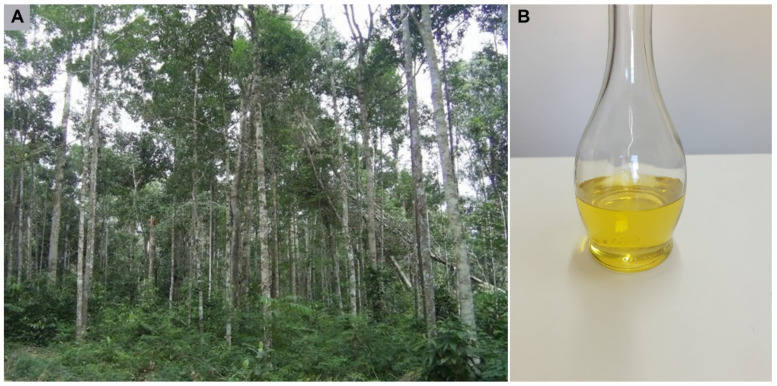
*Aniba rosaeodora* tree (**A**) and essential oil (**B**).

*Aniba rosaeodora* Ducke, Lauraceae is a large tree that reaches 30 m in height, with yellow-brown bark (hence the name rosewood) and grows in the Amazon region (Figure 1A) [5]. Rosewood essential oil (Figure 1B) is widely used in the perfumery industry and its extraction for industrial purposes began in the interior of the state of Pará, Brazil, around 1930 [6]. All parts of the tree are fragrant, although only the trunk wood is harvested and hydrodistilled to obtain rosewood oil, a valuable product. The species has been used in the Amazon as a medicinal plant to control epileptic seizures, to compose regional fragrances and as an ornamental plant [7].

Rosewood oil is a colorless to pale yellow liquid with a woody floral fragrance, containing monoterpenic alcohol linalool as the main constituent. It is of interest to the flavor and fragrance industries because it is transformed into several valuable derivatives [8]. There are few reports of pharmacological properties of Rosewood oil associated with its chemical profile. These properties are the result of the synergism of all the molecules present in the oil or reflect the activity of its major compound linalool, presenting antibacterial and antifungal [9,10], antioxidant [11] and antiprotozoan properties [12].

Available data on the biological activity of rosewood essential oil are quite limited, because most studies have focused on linalool, its main constituent [13]. Thus, this work aims to evaluate the chemical composition of *Aniba rosaeodora* essential oil extracted in the Amazon biome, as well as its antibacterial, antioxidant and trypanocidal activity.

## 2. Results

### 2.1. Physical and Chemical Characterization of A. rosaeodora Essential Oil

**Figure 2:**
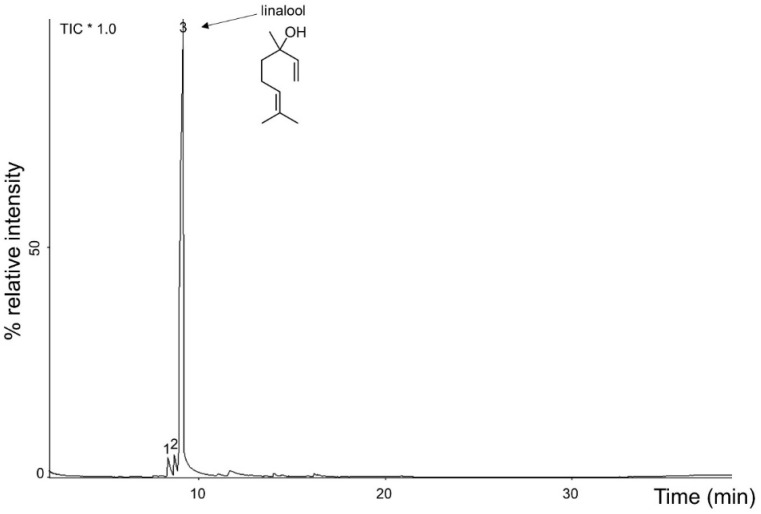
Chromatogram of *Aniba rosaeodora* essential oil. * TIC: total ion chromatogram.

The yield of *A. rosaeodora* essential oil obtained from dried leaves thin branches was 2.8%. The oil showed a yellow color and a clean appearance (Figure 1B), and presented a density of 0.89 g/mL at 25 °C, and a refractive index (ND 25) of 1.459. It was soluble in ethanol 70% in a ratio of 1:2. Chemical compounds identified and quantified in *A. rosaeodora* essential oil are presented in the chromatogram (Figure 2) and Table 1. Three compounds were identified and enumerated accordingly with elution order and retention time. The major constituent of *A. rosaeodora* essential oil was linalool with 93.60%. In addition, α-terpinolene and linalool cis-oxide were identified and quantified at 3.37% and 3.03%, respectively.

### 2.2. Antimicrobial Activity of A. rosaeodora Essential Oil and Linalool

Bacteria from the marine environment were evaluated by the disc-diffusion method against *A. rosaeodora*, linalool and several reference antibiotics (Table 2). The bacteria culture displayed inhibition halos ranging from 7 to 25 mm, besides the non-inhibition presented in some cultures. Comparing linalool with *A. rosaeodora* essential oil, we found that *A. rosaeodora* was more efficient against *Aeromonas caviae* and *Enterococcus faecalis* than the standard linalool. Linalool exhibited greater activity against *Klebsiella pneumonia* and *Providencia stuartii* than *A. rosaeodora* essential oil, while both compounds presented the same activity against *Aeromonas hydrophila*. The susceptibility test performed with antibiotics showed that *E. faecalis* was the strain that presented sensibility for all the antibiotics analyzed, while the other four strains displayed mixed sensibility to the antibiotics.

The preliminary antibacterial activity against standard strain bacteria evaluated by the disc-diffusion method showed a growth inhibitory halo on *A. rosaeodora* essential oil and linalool disks against Gram-positive (*Staphylococcus aureus*) and Gram-negative strains (*Escherichia coli*, *Pseudomonas aeruginosa* and *Salmonella* sp.) (Table 3). Gram-positive bacteria exhibited the highest inhibition halo from both essential oil and linalool. The minimum inhibitory concentration (MIC) of *A. rosaeodora* essential oil ranged from 250 to 450 μg/mL against the tested strains. As observed in the disc-diffusion method, *S. aureus* was the strain more sensible to *A. rosaeodora* essential oil and linalool activity by MIC analysis. Likewise, both methodologies showed that *A. rosaeodora* essential oil presented better antimicrobial activity than linalool to all strains analyzed.

### 2.3. Antioxidant Activity of A. rosaeodora Essential Oil and Linalol

**Figure 3:**
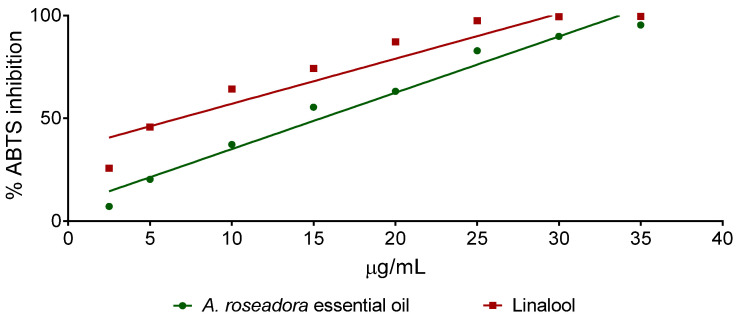
Inhibition of the 2,2-azinobis- (3-ethylbenzothiazoline-6-sulfonic acid) (ABTS) radical by *Aniba rosaeodora* essential oil and linalool.

*Aniba roseadora* essential oil and linalool presented antioxidant activity concentration-dependent, as observed in the graph that relates *A. rosaeodora* essential oil and linalool concentration versus the percentage of inhibition of the 2,2-azinobis- (3-ethylbenzothiazoline-6-sulfonic acid) (ABTS) radical (Figure 3). The calculated EC_50_ was 15.46 µg/mL for *A. rosaeodora* essential oil and 6.78 µg/mL for linalool.

### 2.4. Cytotoxicity, Antitrypanosomal Activivy and Selectivity Index of A. rosaeodora Essential Oil and Linalool

**Figure 4:**
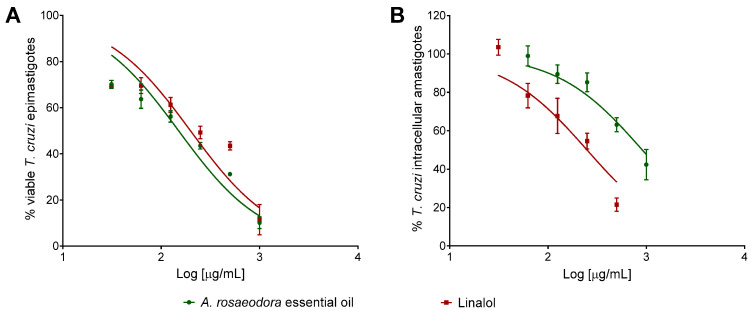
Activity of *Aniba rosaeodora* essential oil and linalool against *Trypanosoma cruzi* epimastigote (**A**) and intracellular amastigote forms (**B**) after 24 h of treatment. Data represent media ± standard deviation of three independent experiment realized in triplicate.

The activity of *A. rosaeodora* essential oil and linalool was evaluated against the epimastigote and intracellular amastigote forms of *Trypanosoma cruzi*, as well as its cytotoxic effect against mammal cells. Both compounds presented concentration-dependent inhibitory activity against epimastigote and intracellular amastigote forms of *T. cruzi* (Figure 4). The inhibitory concentration for 50% of parasites (IC_50_) values for epimastigote forms was lower for *A. rosaeodora* essential oil than linalool. Analyzing the activity against different forms of the *T. cruzi*, *A. rosaeodora* essential oil exhibited IC_50_ value against epimastigote 6.0-fold higher in comparison to the IC_50_ against intracellular amastigotes forms. In contrast, linalool was 3.65-fold more effective against *T. cruzi* intracellular amastigote when compared to *A. rosaeodora* essential oil. Both compounds presented higher IC_50_ values when compared to benznidazole. Cytotoxicity assay revealed that *A. roseadora* essential oil and linalool not showed toxicity for BALB/c peritoneal macrophages even at the highest concentration analyzed (1000 μg/mL). Thus, linalool exhibited higher SI value than *A. roseadora* essential oil (Table 4).

**Figure 5:**
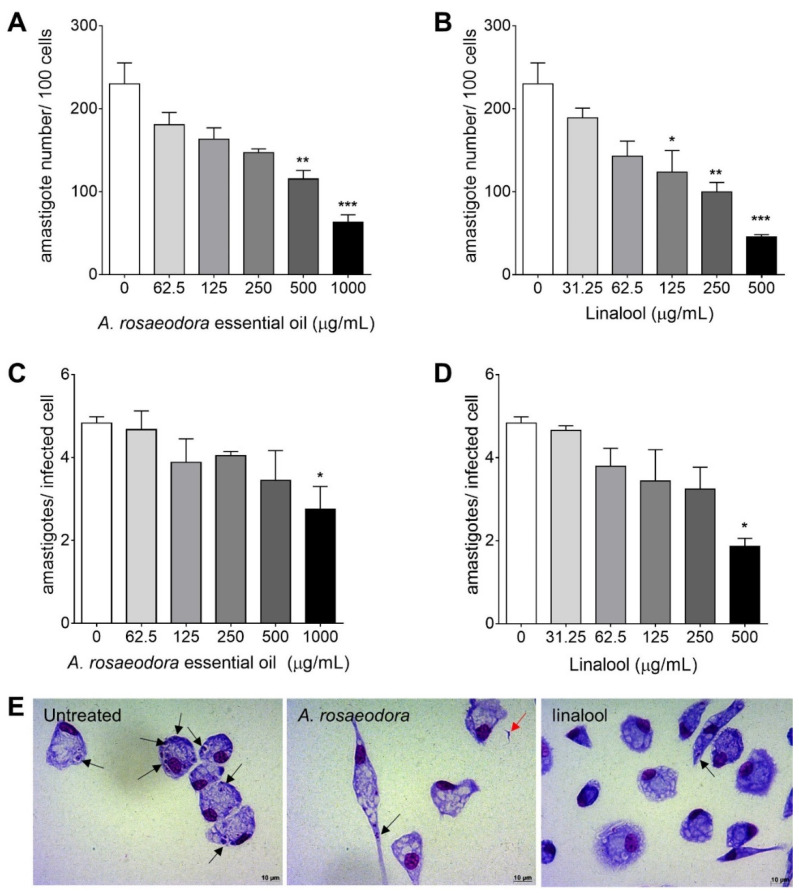
BALB/c peritoneal macrophages infected with *Trypanosoma cruzi* and treated for 24 h with *Aniba rosaeodora* essential oil or linalool. (**A**–**D**) Parameters of infection and (**E**) light microscopy after *A. rosaeodora* or linalool treatment at 1000 or 500 μg/mL respectively. Intracellular amastigotes inside macrophages (black arrows) and non-internalized parasite (red arrows). The images and data (mean ± standard deviation) represent two independent experiments performed in quadruplicate. * *p* < 0.05, ** *p* < 0.01 and *** *p* < 0.001 when compared with untreated infected cells by Kruskal–Wallis and Dunn’s multiple comparison test. Giemsa, 40× objective.

The parameters of infection analysis (Figure 5) showed that *A. rosaeodora* essential oil treatment displayed significant low number of amastigotes per 100 cells at 1000 μg/mL (*p* = 0.0001) and 500 μg/mL (*p* = 0.0011) (Figure 5A). Linalool showed a low number of amastigotes per 100 cells at 500 μg/mL (*p* = 0.0001), 250 μg/mL (*p* = 0.0014) and 125 μg/mL (*p* = 0.0290) (Figure 5B). On the other hand, the treatment with *A. rosaeodora* essential oil and linalool only presented a significant low mean number of amastigotes per infected cells at 1000 μg/mL (*p* = 0.0398, Figure 5C) and 500 μg/mL (*p* = 0.0229, Figure 5D), respectively. The alterations in intracellular amastigotes of *T. cruzi* after treatment with *A. rosaeodora* essential oil and linalool are represented in photomicrography images of Figure 5E.

### 2.5. Nitrite Quantification in T. Cruzi-Infected Peritoneal Macrophages Treated with A. rosaeodora Essential Oil and Linalool

**Figure 6:**
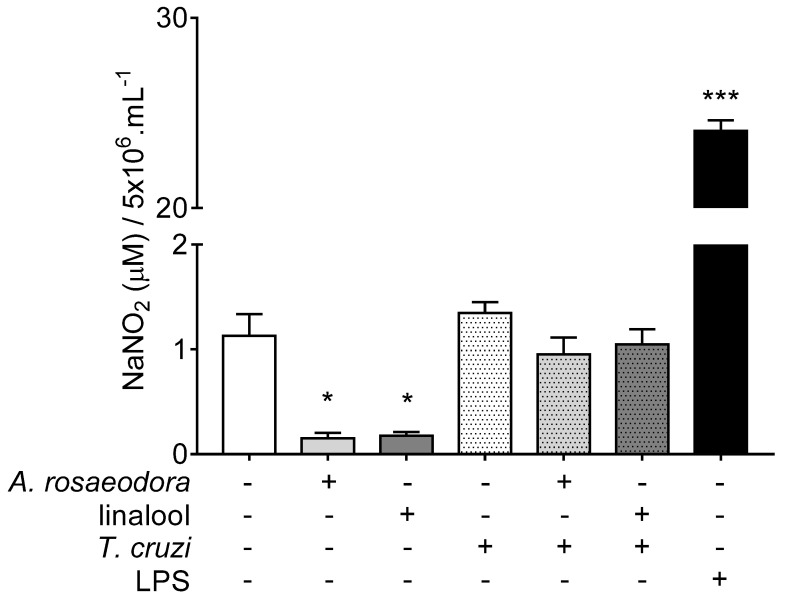
Nitrite quantification in the supernatant of the BALB/c peritoneal macrophage treated with *Aniba rosaeodora* essential oil (500 µg/mL) or linalool (125 µg /mL), and stimulated or not with *Trypanosoma cruzi*. Data represents mean ± standard deviation of experiment realized in sextuplicate; * *p* < 0.05, *** *p* < 0.001 when compared with untreated and unstimulated macrophages by Kruskal–Wallis and Dunn’s multiple comparison test.

The nitrite quantification in the supernatant of BALB/c peritoneal macrophages showed low nitrite levels in cells treated with *A. rosaeodora* essential oil (0.150 ± 0.220 μM NaNO_2_, *p* = 0.0259) and linalool (0.175 ± 0.146 μM NaNO_2_, *p* = 0.0490) when compared to untreated-unstimulated cells (1.129 ± 0.501 μM NaNO_2_). In *T. cruzi*-stimulated cells, although nitrite levels after treatment with *A. rosaeodora* essential oil (0.952 ± 0.779 μM NaNO_2_) and linalool (1.047 ± 0.702 μM NaNO_2_) were lower than stimulated-untreated cells (1.347 ± 0.416 μM NaNO_2_), the difference was not statistically significant for both compounds (*p* = 0.945 and *p* > 0.999, respectively) (Figure 6).

## 3. Discussion

Essential oil may change depending on the chemical nature of its constituents and can be modified by air, light, heat, water and various impurities of natural origin or from falsifications. The changes can be recognized both by changes in their organoleptic characteristics (aroma, color, taste, transparency, fluidity), as well as the values of their chemical and physical parameters. Thus the density, refractive index, solubility, color and appearance were analyzed and the physical characteristics of the essential oil were similar to the pattern described in previous studies of *A. rosaeodora* [14].

Studies has identified and quantified chemical compounds of *A. rosaeodora* essential oil, revealing that this species has chemotypes similar to essential oil extracted in Belém, state of Pará, Brazil, with linalool (84.8%) as the major compound, followed by α-terpineol (2.9%), geraniol (1.0%), benzyl benzoate (0.6%) and minimal amounts of monoterpene hydrocarbons and oxygenated sesquiterpenes (9.2%) [15]. The same was observed in the study of *A. rosaeodora* essential oil extracted in São Paulo, Brazil, where the presence of linalool (81.45%), trans-linalool oxide (1.19%), R-terpineol (1.09%) were observed [16]. Almeida et al. (2013) also reported that linalool is the main compound in essential oil obtained from wood, leaves and branches of the Brazilian rosewood [17].

The disk diffusion test carried out against standard strain bacteria and against bacteria isolated from a marine environment evidenced antibacterial activity, preliminarily. The mixed sensibility observed to the several antibiotics revealed the resistance pattern of marine environment bacteria. The sensibility observed to *A. rosaeodora* essential oil and linalool showed that both compounds have activity against marine environment bacteria used in this study. It is known that the bacterial cell wall influences in an important way on the action of certain antibiotics. The difference between Gram-positive and Gram-negative bacterial walls would be one of the responses to antibiotic resistance between two bacteria [14]. Bacterial resistance is best evidenced in environmental bacteria in the last few years. With the advent of modernization, an increasing amount of antibiotics was released into the environment along with the residues from domestic, industrial, agricultural and medical activities. This has ended up generating a selection of antibiotic-resistant bacteria or genes in the environment, which threatens the efficiency of antibiotics in fighting bacterial infections [18].

The MIC of *A. rosaeodora* essential oil resulted in concentrations lower than linalool. Holetz, et al. (2002) classifies samples that have MIC values below 100 μg/mL with good antibacterial activity; 100 to 500 μg/mL moderate; and 500 to 1000 μg/mL weak above 1000 μg/mL inactive [19]. Following this classification, *A. rosaeodora* essential oil showed moderate activity, while linalool displayed weak activity. The difference between the activity of both compounds may be related to the synergistic effect of the compounds present in the essential oil of *A. rosaeodora*. A synergistic interaction can be verified between the essential oils of *A. rosaeodora* and *Pelargonium graveolens* with gentamicin, and a very strong synergistic interaction against *Acinetobacter baumannii* ATCC 19606 (fractional inhibitory concentration/FIC index = 0.11) [20]. While research conducted with linalool showed low activity against Gram-positive and Gram-negative bacteria. Jabir et al. (2018) found that linalool loaded in gold nanoparticles modified with glutathione (LIN-GNPs) has effective antibacterial activity against Gram-positive bacteria, proving that LIN-GNPs acted on the bacterial cell membrane, in giving up and increasing cell wall permeability and stimulated reactive oxygen species (ROS) production that leads to bacterial nucleic acid damage [21].

Linalool is a compound widely used by the cosmetics industry [22] In the study by Herman et al. (2016) [10], a significant increase in antimicrobial efficacy was observed by the addition of linalool to essential oil, reducing its concentrations in products (cosmetics, medicine), making it possible to obtain its synergistic and additive effects. In addition, several studies have been reported on the commercial availability of oxidized linalool samples possibly causing allergic contact dermatitis [23,24,25]. Thus, essential oil from *A. rosaeodora* has potential applicability in edible and/or dermatological preparations.

Biological activity may be directly related to phenolic compounds as they are good electron donors and therefore have efficient antioxidant activity among secondary plant metabolites. These compounds are capable of control oxidative damage generated by reactive oxygen species or radicals [26]. We can also classify the antioxidant activity according to the excellent (IC_50_ < 15 µg/mL), good (15 µg/mL < IC_50_ < 50 µg/mL), medium (50 µg/mL < IC_50_ < 100 µg/mL), and weak activity (IC_50_ ≥ 100 µg/mL). *A. rosaeodora* essential oil antioxidant activity was considered good while the linalool was optimal, corroborating with previous studies that verified excellent antioxidant activity of *Aniba* species [26].

In traditional medicine, plant essential oils are known as a rich source of secondary metabolites with relevant biological activities, as an alternative in antiparasitic therapy [27,28]. The trypanocidal activity of essential oils of *Aniba* genus was described in the literature [14,29]. Currently, the drugs available for the treatment of Chagas disease are benznidazole and nifurtimox, which have limited efficacy, serious adverse effects and have been in use since the late 1960s [30]. Thus, in an attempt to search for new therapeutic alternatives for Chagas disease, we report the effect of *A. rosaeodora* essential oil and its main component linalool in the growth of epimastigote and intracellular amastigote forms of *T. cruzi*.

In the present study, *A. rosaeodora* essential oil showed activity against epimastigote forms. Literature data showed anti-*T. cruzi* activity in vitro in extracts and substances of different species of the *Aniba* genus of plants collected in the Amazon [29], with promising antileishmanial activity [14]. To understand whether linalool is responsible for the inhibitory activity, an analysis of linalool against epimastigote was performed. The results showed an inhibitory effect close to the values of *A. rosaeodora* essential oil. Therefore, it is worth inferring that the inhibitory effect of the essential oil occurs due to the high concentration of linalool, or due to a possible synergistic and/or additive effect of the constituents of the essential oil acting as trypanocidal agents [31].

Previous data demonstrated that the IC_50_/24 h for linalool was 162.5 μg/mL for epimastigotes and 264 μg/mL for *T. cruzi* trypomastigotes (Y strain) [32], corroborating with data presented in this study. However, linalool had a potent trypanocidal effect against the trypomastigote form of *T. cruzi* (clone Dm28c) derived from cells, with IC_50_/24 h of 306 ng/mL, indicating that different forms and/or origin and different strains may differ in their susceptibility to essential oil derivatives [33].

The search for new therapeutic drugs requires conditions that simulate the environment found by the parasite–cell interaction, therefore, the assay against intracellular amastigote forms of tripanosomatids may represent ideal conditions, with macrophages playing an important role in the evaluation of drug-mediated toxicity [34]. Thus, it was evaluated whether *A. rosaeodora* essential oil and linalool could inhibit *T. cruzi* intracellular amastigote. However, the inhibitory effect was observed only when infected cells were treated with linalool although in high concentration, while *A. rosaeodora* essential oil presented activity at an even higher concentration.

*Piper aduncum* essential oil (PaEO), with nerolidol (25.22%) and linalool (13.42%) as main constituents, effectively inhibits the intracellular survival/replication of T. cruzi amastigotes. PaEO at a concentration of 12.5 µg/mL decreased the rate of T. cruzi amastigote infection by 71.5%, with an IC_50_/24 h of 9 µg/mL. As linalool showed trypanocidal activity, with IC_50_/24 h of 306 ng/mL against trypomastigotes [33], it is possible to infer that activity against intracellular amastigote forms it is possibly due to linalool presence. In addition, previous data demonstrated that at low concentrations of purified linalool derived from the *Croton cajucara* essential oil, the number of parasites internalized in the macrophages decreased (treated before and after the interaction). On the other hand, no cytotoxic effects of essential oil and linalool were observed in peritoneal macrophages of Swiss mice and Vero cells [35]. As in our study, *A. rosaeodora* essential oil and linalool not exhibited cytotoxicity against peritoneal macrophages in the concentration range under analysis. As a result, linalool showed a select activity to the parasites when compared to mammalian cells [14].

Literature data with *L. infantum chagasi* determined that the post-interaction treatment with linalool has antiparasitic activity against intracellular amastigotes, inducing a decrease in the number of parasites within the macrophages [36]. In the same study, it was observed that linalool is capable of providing a drastic change in oxygen consumption, probably related to mitochondrial dysfunction. *P. aduncum* essential oil rich in linalool induced mitochondria dysfunction altering the mitochondrial membrane potential of the T. cruzi epimastigote [33]. Mitochondrial alterations as swelling and important changes in the organization of nuclear and kinetoplastic chromatins were observed by electron microscopy when *L. amazonensis* parasites were treated with *C. cajucara* essential oil [35]. Linalool may interfere with the integrity of protozoan mitochondria, however, further studies are needed to elucidate the mechanism involved in the trypanocidal activity observed in our study.

An indirect mechanism involved with antitrypanosomal activity is related with macrophage activation, particularly the nitric oxide (NO) induction. The NO-mediation directly kills *T. cruzi* in vitro [37]. Thus, we carried out an analysis of the nitrite quantification of *T. cruzi*-stimulated peritoneal macrophages treated with *A. rosaeodora* essential oil or linalool. However, a significant decrease in nitrite levels was observed in cells non-stimulated with *T. cruzi* and treated with *A. rosaeodora* essential oil or linalool. Reactive oxygen species decrease was also observed in cancer cells lines treated with *A. rosaeodora* essential oil, inhibiting apoptosis in these cells [13].

Otherwise, in *T. cruzi*-stimulated cells the treatment with *A. rosaeodora* essential oil or linalool did not significantly decrease nitrite levels. Linalool has a known anti-inflammatory activity [38] and inhibits NO formation in vitro [39], but interestingly, an in vitro experiment of macrophages treated with linalool (250 or 350 µg/mL) for 24 h before or after interactions with the *Leishmania infantum* was also not associated with any difference in NO production [36]. The inhibition of NO production observed in macrophages treated with *A. rosaeodora* essential oil and linalool, although it is not associated with antitrypanosomal activity, is an interesting finding that should be better elucidated in further studies.

## 4. Materials and Methods

### 4.1. Reagents

Anhydrous sodium sulfate, ethanol, ethyl acetate, dimethyl sulfoxide (DMSO), eugenol, 2,2′-azino-bis(3-ethylbenzothiazoline-6-sulfonic acid) diammonium salt (ABTS), penicillin, streptomycin, N-benzyl-2-nitro-1H-imidazole-1-acetamide (Benznidazole), Brewer thioglycolate medium, RPMI 1640 medium, 3-(4,5-Dimethyl-2-thiazolyl)-2,5-diphenyl-2H-tetrazolium bromide (MTT), sulfanilamide, H_3_PO_4_, N-(1-naphthyl)ethylenediamine and sodium nitrite were purchased from Sigma, St Louis, MO, USA. Giemsa’s azur-eosin-methylene blue, Brain Heart Infusion broth, Mueller-Hinton agar and Mueller-Hinton broth were purchased from MERK, Darmstadt, Germany. The other bacteria culture medium were purchased from BD, Becton Dickinson, Franklin Lakes, NJ, USA. API^®^ 20 E system was purchased from bioMérieux, Durham, NC, USA. Fetal bovine serum (FBS) was purchased from Gibco, Gaithersburg, MD, USA.

### 4.2. Plant Material

Authentic samples of the *A. rosaeodora* species were obtained from three trees cultivated at the Adolpho Ducke Forest Reserve, Highway AM-010, km 26 (latitude −2.908185, longitude −59.975457), Manaus, Brazil. Leaves and thin branches were harvested with a trimmer from the treetops in the dry season, March 2017. The taxonomic identification was undertaken by the Herbarium of the Department of Botany of the Universidade Federal do Amazonas, registry number 5982. The leaves were selected and dried in an oven at 37 °C for 48 h and sprayed in an electric knife mill at the Food and Water Quality Control Laboratory of the Federal University of Maranhão.

### 4.3. Essential Oil Extraction

The extraction of the essential oil of *A. rosaeodora* was carried out with 100 g of dried leaves from thin branches diluted in water in the proportion of 1:10 by hydrodistillation using the Clevenger system for 3 h at 100 °C. The essential oil collected were dried with anhydrous sodium sulfate (Na_2_SO_4_) and the final volume found was used to determine the yield through the mass/volume ratio by measuring the density. Mass/volume ratios were calculated from the mass (g) of the initial vegetal material and the volume (mL) of essential oil obtained after extraction. The essential oil samples were kept at 25 °C and then weighed. For the verification of biological activity in vitro, the essential oil and the reference drugs were diluted in DMSO and subsequently made serial dilutions in an appropriate culture medium until reaching a final concentration below 1% DMSO.

### 4.4. Physical-Chemical Analysis of Essential Oil

Physical-chemical analyzes performed on *A. rosaeodora* essential oil were: density, measured with a glass pycnometer; refractive index, calculated with an ABBE 2WAJ refractometer (PCE Instruments, Southampton, UK); the color and appearance, that were visually verified by three different people; and the determination of solubility, carried out through the ratio of 1:1 of oil and 70% ethanol until its complete solubilization.

### 4.5. Gas Chromatography–Mass Spectrometry (GC–MS)

The standard used in the development of the analytical methodology was linalool. Standard solutions of monoterpenes were prepared by dilution in absolute ethyl alcohol and chloroform at different concentrations. The essential oil of *A. rosaeodora* was solubilized in ethyl acetate and was analyzed by a gas chromatograph Shimadzu QP 5000 (Shimadzu Corp., Kyoto, Japan), a column used with a capillary ZB-5 ms (5% phenyl arylene 95% dimethylpolysiloxane) coupled to 70 eV (40–500 Da) HP 5MS electronic impact detector with a transfer temperature of 280 °C. In the analysis, 0.3 μL of ethyl acetate and helium gas (99.99%) were injected at a temperature of 280 °C, using a split mode (1:10) with an initial temperature gradient of 40 to 300 °C.min^−1^, with a chromatographic run that lasted 30 min.

### 4.6. Bacteria from Marine Enviroment

Bacteria strains isolated from the marine environment *Aeromonas caviae, Aeromonas hydrophila, Enterococcus faecalis, Klebsiella pneumoniae* and *Providencia stuartii* were gently provided by the Laboratory of Microbiology of the Water Quality Control Program at the Federal University of Maranhão. Water samples were aseptically collected from approximately 30 cm below the water surface of the Jansen lagoon, Maranhão Brazil (latitude −2.499629, longitude −44.301211). Then, the samples were transported to the Microbiology Laboratory of the Federal University of Maranhão in isothermal boxes containing ice to perform the identification. To *Aeromonas* isolation, successive decimal dilutions of water samples (10^−1^ to 10^−7^) were prepared in alkaline peptone water (APA), with subsequent distribution of 1 mL aliquots in five series of five tubes containing tryptic soy broth (TSB Broth) and 0.1 mL in plates containing the selective medium, agar gelatin phosphate salt (GSP Agar) (duplicates), both with 20 µg/mL of ampicillin, an antibiotic used as an inhibitor of the accompanying microbiota of *Aeromonas*. Colonies suspected of being *Aeromonas* were seeded in tilted BD trypticase soy agar (TSA agar) tubes, followed by incubation at 28 °C for 24 h. After, the cultures on TSA agar were subjected to biochemical tests of oxidase, catalase, gas production from glucose for species identification, indole production, O/129 resistance, amino acid decarboxylation (test on triple sugar agar and iron—TSI agar), motility: nitrate reduction, esculine hydrolysis, Voges–Proskauer (VP) assay, carbohydrate fermentation and growth at 3% and 6% sodium chloride. To Enterobacteriaceae isolation and identification of *Klebsiella pneumoniae* and *Providencia stuartii* in the water samples, initially, 25 mL of each sample were homogenized in 225 mL of brain and heart infusion broth (BHI broth) and incubated in a bacteriological oven at 37 °C for three hours. After the incubation period, the entire inoculum was transferred to 250 mL of broth for *Escherichia coli* and incubated at 37 °C for 24 h. Isolation was performed using selective and differential media, methylene blue eosin agar (EMB agar) and MacConkey sorbitol agar (MCS agar). For the identification of the species, initially five colonies were selected from the selective culture media, small colonies with metallic green or black without gloss in EMB agar and those of intense pink color (positive sorbitol) and yellow (negative sorbitol) in MCS agar. Then, the colonies were isolated in tubes containing TSA agar inclined with subsequent incubation at 37 °C for 24 h. Biochemical identification was performed using conventional tests: indole, simmons citrate, methyl red, VP, malonate, carbohydrate fermentation—sorbitol, rhamnose, mannitol, arabinose, inositol and raffinose, decarboxylation of amino acids lysine and ornithine, motility and H_2_S production in sulfide indole motility (SIM) agar [40] and by the API^®^ 20 E system. For *Enterococcus* research, 9 mL of each sample were diluted in 90 mL of buffered peptide water and incubated for 24 h/35 °C. Subsequently dilutions (10^−1^ to 10^−7^) and the highest dilution were plated on M-*Enterococcus* agar and incubated at 35 °C for 48 h. Brick red colonies were inoculated on TSI agar. Tubes that showed suggestive characteristics were analyzed by acid ramp, acid-base, H_2_S (-), catalase, oxidase, 6% NaCl, glucose and esculin tests, and by the API^®^ 20 E system.

### 4.7. Bacterial Strains and Culture Conditions

To perform the preliminary antimicrobial tests, the standard strains *Escherichia coli* (Migula) Castellani and Chalmers (ATCC^®^ 25922™), *Staphylococcus aureus* subsp. *aureus* Rosenbach (ATCC^®^ 12600™), *Pseudomonas aeruginosa* (Schroeter) Migula (ATCC^®^ 27853™) and *Salmonella enterica* subsp. *enterica* (ex Kauffmann and Edwards) Le Minor and *Popoff serovar* Choleraesuis (ATCC^®^ 12011™) were used. The tests were carried out at the Microbiology Laboratory of the Federal University of Maranhão. The strains were grown in BHI broth for 24 h at 37 °C and the inoculum was adjusted to a cell concentration of 10^8^ colony forming unit (CFU)/mL following the MacFarland scale, recommended by the Clinical and Laboratory Standards Institute [41].

### 4.8. Antimicrobial Assays

During the preliminary test of diffusion in solid medium, 100 μL of inoculum of each bacterium sown on Mueller–Hinton agar plates were used, and on the agar surface, a paper disc impregnated with 50 μL of essential oil of *A. rosaeodora*, standard linalool or reference drugs were added; then the plates were incubated at 35 °C and after 24 h the inhibition zone was measured with a millimeter rule [42]. The MIC was also performed according to the broth dilution methodology performed in triplicate with the same bacteria used in the diffusion tests in solid medium [41]. Initially, serial dilutions were performed resulting in concentrations of 5–1000 μg/mL of *A. rosaeodora* essential oil, linalool or reference drugs and transferred to a test tube containing Mueller-Hinton broth. To each concentration, 100 μL of the microbial suspension containing 1.5 × 10^8^ CFU/mL were added and subsequently incubated at 35 °C for 24 h. It was also reserved control of broth sterility and bacterial growth. After the incubation period, the MIC was determined, being defined as the lowest concentration that visibly inhibited bacterial growth (absence of visible turbidity). To confirm growth inhibition, the broth was subjected to the microbial seeding test of the inoculum on the surface of the plate-count agar.

### 4.9. Antioxidant Assay

Antioxidant activity was assessed using a reaction mixture of 2,2-azinobis- (3-ethylbenzothiazoline-6-sulfonic acid) (ABTS) at 3840 μg/mL with 88 μL of 37,840 μg/mL potassium persulfate solution left in the dark at room temperature for 16 h giving rise to the ABTS radical which was diluted in ethanol to obtain an absorbance of 0.7 to 734 nm. The results were obtained in a dark environment, in which 30 µL of each concentration of essential oil (200 to 15 μg/mL) and eugenol (90 to 5 μg/mL) was transferred in test tubes containing 3.0 mL of the cation radical ABTS and homogenized on a tube shaker, and after 6 min the absorbance of the reaction mixture was read on a spectrophotometer at a length of 734 nm [43]. The analyses were carried out in triplicates and the determination of the activity was demonstrated as percentage of inhibition (% I) of the ABTS radical cation according to the equation: % inhibition = (absorbance of the solution of the radical ABTS—absorbance of the sample)/(solution of ABTS absorbance radical) × 100 [44]. We also verified the efficient concentration or EC50% that represents the concentration necessary to sequester 50% of the ABTS root. The essential oil will be considered active when it has an EC50 < 500 μg/mL [45].

### 4.10. Parasites

Parasite cultures employed in this study were *Trypanosoma cruzi* (SC2005 strain). Trypomastigote forms were obtained from Vero cells infected and used to infect the macrophages. Epimatigote forms were originated from the suspension of cell culture trypomastigotes in 3 mL of liver infusion tryptose (LIT) medium supplemented with 10% fetal bovine serum (FBS), 100 U/mL of penicillin and 100 μg/mL of streptomycin), and incubated in an oven at 28 °C until complete differentiation of parasites.

### 4.11. Anti-Epimastigote Assay

Epimastigote forms of *T. cruzi*, from a 2- to 4-day-old culture were incubated for 24 h in the absence or in the presence of different concentrations (1000–15.625 µg/mL) of *A. rosaeodora* essential oil or linalool, obtained by serial dilutions (1:2), at a final volume of 100 µL per well. The controls were identified as blank (wells without parasites), untreated control (parasites and DMSO 1%) and reference drug (benznidazole). Incubation took place in a 96-wells plate, in a BOD incubator at 28 °C in LIT medium using a parasite concentration of 10^6^ promastigotes/mL. After 24 h, with the aid of the Neubauer chamber and light microscopy [46], viability was evaluated by counting parasites and the results were used to calculate the IC_50_ (50% inhibition of parasite growth) following the formula: IC_50_ = (sample counting)/(control counting) ×100 [47].

### 4.12. Animals and Ethical Statements

BALB/c female mice from 4 to 6 weeks of age were purchased from the Institute of Science and Technology in Biomodels of the Institute of Science and Technology in Biomodels. All procedures were performed in accordance with the National Council for the Control of Animal Experimentation National Council for Animal Experimentation Control—CONCEA) and approved by the Ethics Committee on Animal Care and Utilization (CEUA/IOC—L018/2018).

### 4.13. Peritoneal Macrophage Collection and Culture

Peritoneal macrophages from BALB/c mice were collected after elicited with 3 mL 3% Brewer thioglycollate medium broth injection for 72 h, and maintained in RPMI 1640 medium supplemented with 10% FBS, 100 U/mL of penicillin and 100 µg/mL of streptomycin, overnight at 37 °C and 5% CO_2_.

### 4.14. Cytotoxicity Assay

Peritoneal macrophages (5 × 10^5^ cells/mL) were cultured in 96-well plates with different concentrations, obtained by serial dilutions (1:2), of *A. rosaeodora* essential oil or linalool (1000–7.8 μg/mL) or benznidazole (200–0.78 μg/mL) up to a final volume of 100 μL per well. The controls were categorized as blanks (wells with culture medium without cells), untreated control (cells and DMSO 1%) and reference drug (benznidazole). After 72 h, the cell viability was analyzed by the MTT colorimetric method [48]. Absorbance was measured in a spectrophotometer at 540 nm wavelength. The concentration inhibiting 50% of cell growth (CC_50_) was calculated following the formula: CC_50_ = (sample absorbance-blank absorbance)/(control absorbance-blank absorbance) × 100 [49].

### 4.15. Activitiy Against Intracellular Amastigotes and Selectivity Index (SI)

BALB/c peritoneal macrophages cultured in 24-well plates (5 × 10^5^ cells/well), with coverslips, were infected with trypomastigote forms of *T. cruzi*, obtained from cultured Vero cells, using the ratio of parasite/cell 10:1, at 37 °C and 5% CO_2_ for 6 h. After incubation, well plates were washed with phosphate-buffered saline (PBS, pH 7.2) to remove the non-internalized parasites. The infected cells were treated with different concentrations of *A. rosaeodora* essential oil or linalool (1000–31.25 µg/mL), or benzonidazole (100–6.25 µg/mL) for 24 h. The amastigotes couting by analysis of light microscopy were carried out to determine the IC_50_ calculation. Selectivity index were obtained from the relationship of macrophage cytotoxicity and antiamastigote activity. Parameters of infection analysis were performed according to Teles et al. [50].

### 4.16. Nitrite Quantification

BALB/c peritoneal macrophages (5 × 10^6^ cells/mL) was treated with *A. rosaeodora* essential oil (500 µg/mL) or linalool (250 µg/mL), and either stimulated or not stimulated with *T. cruzi* trypomastigotes (5 × 10^7^ parasites/mL) for 48 h. Nitrite quantification of the supernatant of the cells was performed with Griess reagent. Briefly, 50 µL of culture supernatant and 50 µL of Griess reagent (25 µL of sulfanilamide 1% in 2.5% H_3_PO_4_ solution and 25 µL of N-(1-naphthyl)-ethylenediamine 0.1% solution) were added in 96-well plates. After incubation in a dark environment for 10 min, absorbance was obtained at 570 nm on the spectrophotometer. The nitrite values were obtained from the standard curve of sodium nitrite (100–1.5 µM) [51].

### 4.17. Statistical Analysis

The numerical results from at least two independent assays were expressed as mean ± standard deviation and the IC_50_ and CC_50_ determination were performed with the GraphPad Prism 7.00 software package (GraphPad Software, San Diego, CA, USA). Kruskal-Wallis and Dunn’s multiple comparison test was used to analyze the data and the difference at *p* < 0.05 was considered significant.

## 5. Conclusions

The essential oil of *A. rosaeodora* showed activity against all the strains tested, with a lower minimum inhibitory concentration being observed for *S. aureus*. An efficient antioxidant activity of the essential oil was evidenced by the ABTS radical discoloration technique, fully inhibiting the radical in relatively low concentrations. These results point to an important potential for use as an antimicrobial and antioxidant agent. The antitrypanosomal activity of *A. rosaeodora* essential oil and linalool were observed at high concentrations against epimatigote forms, and even higher against intracellular amastigotes of *T. cruzi*. Both *A. rosaeodora* essential oil and linalool reduced nitrite levels in unstimulated cells revealing a potential effect in NO production. These data revealed the pharmacological potential of the *A. rosaeodora* essential oil and linalool, which encourage further studies.

## Figures and Tables

**Table 1:** Chemical composition of *Aniba rosaeodora* essential oil.

Peak	Compounds	Retention Time (min)	Peak Area (%) ^1^
1	α-terpinolene	8.361	3.37
2	linalool cis-oxide	8.702	3.03
3	linalool	9.177	93.60

^1^ Peak area percentage in relation to peak total area.

**Table 2:** Inhibitory zone diameters of *Aniba rosaeodora* essential oil, linalool and antibiotics on different bacteria isolated from marine environment after 24 h of treatment.

Compounds/Antibiotics	Bacteria Strain				
	*Aeromonas caviae*	*Aeromonas hydrophila*	*Enterococcus faecalis*	*Klebsiella pneumoniae*	*Providencia stuartii*
*A. rosaeodora* essential oil	22.33 ± 0.577	11.33 ± 0.577	21.33 ± 0.577	10.33 ± 0.577	9.33 ± 0.577
linalool	7.33 ± 0.577	11.66 ± 0.577	13.33 ± 0.577	11.66 ± 0.577	11.33 ± 0.577
pipemidic acid	22.33 ± 0.577	21.66 ± 0.577	18.66 ± 0.577	22.33±0.577	22.33 ± 0.577
ampicillin	0 ± 0.00	0 ± 0.00	15.66 ± 0.577	0 ± 0.00	0 ± 0.00
cefotaxime	24.67 ± 0.577	23.66 ± 0.577	17.66 ± 0.577	17.33 ± 0.577	25.33 ± 0.577
cefoxitin	0 ± 0.00	13.66 ± 0.577	22.66 ± 0.577	8.33 ± 0.577	15.33 ± 0.577
chloramphenicol	23.33 ± 1.155	20.66 ± 0.577	24.66 ± 0.577	13.66 ± 0.577	13.33 ± 0.577
erythromycin	0 ± 0.00	0 ± 0.00	25.66 ± 1.527	0 ± 0.00	0 ± 0.00
gentamycin	17.66 ± 0.577	14.66 ± 0.577	22.33 ± 0.577	14.33 ± 0.577	14.33 ± 0.577
lincomycin	0 ± 0.00	0 ± 0.00	29.66 ± 0.577	0 ± 0.00	0 ± 0.00
oxacillin	0 ± 0.00	0 ± 0.00	18.66 ± 0.577	0 ± 0.00	0 ± 0.00
sulfazotrin	21.66 ± 0.577	19.66 ± 0.577	21 ± 1.00	20.33 ± 0.577	21.33 ± 0.577
tetracycline	10.33 ± 0.577	22.66 ± 0.577	19.66 ± 0.577	0 ± 0.00	18.33 ± 0.577
vancomycin	0 ± 0.00	0 ± 0.00	20.66 ± 0.577	0 ± 0.00	0 ± 0.00

Data represents mean ± standard deviation of experiment realized in triplicate.

**Table 3:** Inhibitory zone diameters and minimum inhibitory concentration of *Aniba rosaeodora* essential oil on different bacterial cultures after 24 h of treatment.

Antimicrobial assay	Compounds	Bacteria Strain			
		*Escherichia coli*	*Staphylococcus aureus*	*Pseudomonas aeruginosa*	*Salmonella choleraesuis*
Inhibition zones (mm) by disc-diffusion	*A. rosaeodora*	15.0 ± 0.00	18.0 ± 0.00	13.0 ± 0.00	14.0 ± 0.00
	linalool	13.0 ± 0.00	15.0 ± 0.00	12.0 ± 0.00	12.0 ± 0.00
	gentamycin	14.0 ± 0.00	20.5 ± 0.70	17.0 ± 0.00	n.d.
	penicillin	n.d.	n.d.	n.d.	18.5 ± 0.70
MIC (μg/mL)	*A. rosaeodora*	350.0 ± 0.00	250.0 ± 0.00	450.0 ± 0.00	400.0 ± 0.00
	linalool	650.0 ± 0.00	550.0 ± 0.00	650.0 ± 0.00	650.0 ± 0.00
	amoxicillin	16.0 ± 0.00	8.0 ± 0.00	n.d.	n.d.
	gentamycin	n.d.	2.0 ± 0.00	n.d.	8.0 ± 0.00
	polymyxin B	n.d.	n.d.	16.0 ± 0.00	n.d.

MIC: minimum inhibitory concentration; n.d.: not determined. Data represents mean ± standard deviation of experiment realized in triplicate.

**Table 4:** BALB/c peritoneal macrophage cytotoxicity, trypanocidal activity and selectivity index of *Aniba rosaeodora* essential oil and linalool.

*Essential oil*/Compounds	Citotoxicity CC_50_ (µg/mL)	*T. cruzi* IC_50_ (µg/mL)		SI
	Peritoneal Macrophage	Epimastigote	Intracellular Amastigote	
*A. rosaeodora*	>1000	150.5 ± 1.08	911.6 ± 1.15	>1.0
linalool	>1000	198.6 ± 1.12	249.6 ± 1.18	>4.0
benznidazole	162.0 ± 1.11	1.805 ± 1.13	0.4820 ± 1.17	336.0

IC_50_: inhibitory concentration for 50% of parasites; CC_50_: cytotoxic concentration for 50% of cells; SI: selectivity index, obtained from the ratio CC_50_/IC_50_ intracellular amastigote. Data represents mean ± standard deviation of at least two independent experiments carried out in triplicate.

## Data Availability

All datasets presented in this study are included in the article.
